# An engineered miRNA PS-OMe miR130 inhibits acute lung injury by targeting eCIRP in sepsis

**DOI:** 10.1186/s10020-023-00607-8

**Published:** 2023-02-13

**Authors:** Timothy Borjas, Asha Jacob, Molly Kobritz, Gaifeng Ma, Chuyi Tan, Vihas Patel, Gene F. Coppa, Monowar Aziz, Ping Wang

**Affiliations:** 1 Department of Surgery, Zucker School of Medicine at Hofstra/Northwell, Manhasset, NY USA; 2 Department of Molecular Medicine, Zucker School of Medicine at Hofstra/Northwell, Manhasset, NY USA; 3 Center for Immunology and Inflammation, The Feinstein Institutes for Medical Research, 350 Community Drive, Manhasset, NY USA

**Keywords:** Sepsis, eCIRP, miRNA, eCIRP inhibitor, Inflammation, Apoptosis

## Abstract

**Background:**

Sepsis is caused by the dysregulated immune response due to an initial infection and results in significant morbidity and mortality in humans. Extracellular cold inducible RNA binding protein (eCIRP) is a novel mediator identified in sepsis. We have previously discovered that microRNA 130b-3p inhibits eCIRP mediated inflammation. As RNA mimics are very unstable in vivo, we hypothesize that an engineered miRNA 130b-3p mimic named PS-OMe miR130, improves stability of the miRNA by protection from nuclease activity. We further hypothesize that PS-OMe miR130 reduces not only eCIRP-mediated inflammation and but also acute lung injury in a murine model of polymicrobial sepsis.

**Methods:**

Single stranded PS-OMe miR130 was synthesized and the binding affinity to eCIRP was evaluated using surface plasmon resonance (SPR) and computational modeling. Macrophages were treated with PS-OMe miR130 with and without eCIRP and cell supernatant analyzed for cytokines. In vitro stability and the in vivo half-life of PS-OMe miR130 were also assessed. The effect of PS-Ome miR130 on eCIRP’s binding to TLR4 was evaluated by SPR analysis and modeling. Finally, the effect of PS-OMe miR130 on inflammation and injury was assessed in a murine model of sepsis.

**Results:**

We demonstrate via SPR and computational modeling that PS-OMe miR130 has a strong binding affinity to eCIRP. This engineered miRNA decreases eCIRP induced TNF-α and IL-6 proteins, and it is highly stable in vitro and has a long in vivo half-life. We further demonstrate that PS-OMe miR130 blocks eCIRP binding to its receptor TLR4. Finally, we show that PS-OMe miR130 inhibits inflammation and lung injury, and improves survival in murine sepsis.

**Conclusion:**

PS-OMe miR130 can be developed as a novel therapeutic by inhibiting eCIRP-mediated inflammation and acute lung injury in sepsis.

## Introduction

Sepsis is defined as an end-organ injury caused by the dysregulated host response resulting from the initial insult that causes significant morbidity and mortality worldwide (Fleischmann et al. 2016; Seymour et al. 2016; Shankar-Hari et al. 2016). In fact, sepsis affects more than 1.7 million adults in the United States annually, and potentially contributes to over 250,000 deaths annually (Rhee et al. 2019). Further, sepsis presents a significant cost to hospital systems as it is widely prevalent and requires intensive treatment (Lagu et al. 2012). Importantly, sepsis patients can develop significant hypotension and associated organ injury (Gotts and Matthay 2016). In addition to treating the underlying cause of sepsis using antibiotics and providing supportive care, including fluid resuscitation and vasopressors, it is important to be able to target the dysregulated immune response. As of now, there is still an unmet need to develop therapeutics that target the dysregulated immune response in both the acute and subacute phases of sepsis.

Cold-inducible RNA-binding protein (CIRP) is a 172 amino acid protein that belongs to the family of cold shock proteins (Aziz et al. 2019; Nishiyama et al. 1997; Qiang et al. 2013). Intracellularly, CIRP acts to stabilize mRNAs in different stress conditions to the cell, which include hypothermia and UV radiation (Aziz et al. 2019). Recently our lab discovered that when CIRP becomes extracellular (eCIRP), it functions as a damage-associated molecular pattern (DAMP) and promotes tissue injury and inflammation (Qiang et al. 2013). CIRP is released from the cell during different states of acute inflammation including polymicrobial sepsis, hemorrhagic shock, and different ischemia/reperfusion injuries (Qiang et al. 2013; Borjas et al. 2022; Cen et al. 2016; Denning et al. 2020). Further, in sepsis, eCIRP has been found to exaggerate systemic inflammation, and promote end-organ injury including acute lung injury and acute kidney injury (Zhang et al. 2018). eCIRP causes inflammation via binding to the TLR4 or TREM-1 receptors (Qiang et al. 2013; Denning et al. 2020; Godwin et al. 2015).

As CIRP is an RNA binding protein, we reasoned that CIRP could interact with extracellular circulating RNA including microRNAs. Our lab discovered that the microRNA (miRNA) 130b-3p was found to be elevated in the serum during sepsis in both mice and humans (Gurien et al. 2020). When studying the miRNA in relation to eCIRP, it was discovered that miRNA 130b-3p mimic directly inhibited recombinant murine (rm)CIRP mediated inflammation both in vitro and in vivo, and had a strong binding interaction with eCIRP. This study introduced the novel concept of a possible endogenous miRNA-based inhibitor and regulator of eCIRP’s proinflammatory function in the extracellular space. miRNAs have recently been discovered to be highly stable in blood and are used as markers of disease as they are bound to RNA binding proteins or exosomes; however, oligonucleotides as exogenously administered therapeutics present significant stability and clearance issues for their in vivo use due to their extreme short half-life in circulation (Mitchell et al. 2008; Segal et al. 2020).

There are different strategies to improve the stability of oligonucleotides which include different chemical modifications and delivery systems (Behlke and Behlke 2008; Dasgupta et al. 2021; Dias and Stein 2002; Khvorova et al. 2017; Lennox et al. 2011). Two important oligonucleotide chemical modifications include phosphorothioate bonds (PS) and 2′*O*-methyl ribose modifications (2′OMe). These modifications have been found to improve oligonucleotide stability by protection from nuclease activity (Behlke and Behlke 2008; Khvorova et al. 2017; Lennox et al. 2011; Fu et al. 2019). As RNA mimics are unstable after in vivo administration, we hypothesized that an engineered miRNA 130b-3p mimic using PS and 2′OMe modifications improves the stability and half-life of the miRNA 130b-3p (named PS-OMe miR130). We further hypothesize that PS-OMe miR130 decreases eCIRP-mediated inflammation in vitro and reduce inflammation and tissue injury in a murine model of sepsis. To investigate this, we synthesized the PS-OMe miR130 and employed in vitro studies in macrophages to understand the relationship between eCIRP and the PS-OMe miR130, tested the stability of PS-OMe miR130 in vitro and in vivo, and examined its therapeutic potential in a murine model of polymicrobial sepsis.

## Materials and methods

### Experimental animals

Adult male Sprague–Dawley rats (300 g) and C57BL/6 mice (20–25 g) were purchased from Charles River Laboratories (Wilmington, MA), and housed in a temperature-controlled room on a 12-h light-dark cycle and fed a standard rodent chow diet. Animals were acclimated to the environment for 5–7 days. Every attempt was made to limit the number of animals used. All animal experiments were approved by the Institutional Animal Care and Use Committee (IACUC) of the Feinstein Institutes for Medical Research and were performed in accordance with the National Institutes of Health and the Guide for the Care and Use of Laboratory Animals.

### PS-OMe miR130 synthesis

Single stranded PS-OMe miR130 (5′-mC*mA*mG*mUmGmCmAmAmUmGmAmUmGmAmAmAmGmGmG*mC*mA*mU-3′) was synthesized by Integrated DNA Technologies (Coralville, Iowa) and provided as a lyophilized powder. * Indicates a PS bond and “m” indicates 2′OMe modification. The powder was then resuspended in nuclease free PBS for the desired concentration. A cy-3 labeled PS-OMe miR130 was synthesized and labeled at the 3′ end for the in vivo half-life experiment.

### Treatment of macrophages with eCIRP and PS-OMe miR130

Mouse macrophage cell line RAW264.7 cells were purchased from American Type Culture Collection (ATCC). Peritoneal macrophages were isolated from C57BL6 mice. All cells were cultured in Dulbecco’s modified Eagle medium (DMEM; Life Technologies Corporation, Grand Island, NY) with 10% of heat-inactivated fetal bovine serum (FBS; MP Biomedicals, Santa Ana, CA), 100 U/ml of each penicillin and streptomycin (Thermo Fisher Scientific, Waltham, MA), and 5% glutamine (Life Technologies Corporation). Prior to experiments, the medium was changed to OPTI-MEM (Life Technologies Corporation) for a total of 1 h. Cells were treated for 24 h with 1 µg/ml of eCIRP with or without 10 nM PS-OMe miR130 combined 30 min prior to stimulation of RAW 264.7 cells or peritoneal macrophages. Cell culture supernatant was collected for analysis. eCIRP was produced in house and quality control assays were performed as previously described (Qiang et al. 2013).

### In vitro stability of PS-OMe miR130

8 µM of the PS-OMe miR130 was incubated in DMEM containing 10% non-heat-inactivated FBS, which has nucleases activities including RNAses, for different time points including 0, 6, 24, 48, and 72 h to measure the stability of PS-OMe miR130, similar to other groups (Barragan-Iglesias et al. 2018; Lennox et al. 2010). The control sample was 8 µM of PS-OMe miR130 in DMEM without the 10% non-heat-inactivated FBS. At each time point, an equal volume of 2X TBE-urea sample buffer (Invitrogen, Thermo Fisher Scientific) was added to the sample, and then flash frozen over dry ice. Samples were thawed and heated to 70 °C for 3 min and subsequently run on a 15% polyacrylamide TBE-urea gel (Invitrogen, Thermo Fisher Scientific) for 75 min at 180 V. After, the gel was stained with ethidium bromide (2 µg/ml) for 20 min (Sigma Aldrich, St Louis, MO), and washed with nuclease free water. The gel was imaged on a Bio-Rad gel reader.

### In vivo half-life of PS-OMe miR130

Sprague-Dawley rats (n = 4) underwent induction of anesthesia with 2–4% inhalation isoflurane after which the bilateral groins were shaved and disinfected by swabbing with Betadine alternated two times with 70% alcohol. At this time, the right femoral artery and left femoral vein were cannulated with PE-50 polyethylene tubing (BD, Sparks, MD) containing a small amount of heparin (2 IU/ml) in normal saline solution. At time 0, 125 µl of 100 µM cy-3 labeled PS-OMe miR130 diluted in 375 µl of normal saline was injected via the femoral vein. At different time points, which included every 3 min for the first 15 min, and then every 15 min until 180 min, 200 µl of blood was withdrawn from the femoral artery. Animals were resuscitated at each time point with 200 µl of normal saline via the femoral vein. Serum was collected and fluorescence (550 nm excitation, 570 nm emission) was measured for each sample. The β-half-life (elimination half-life) was then calculated.

### Surface plasmon resonance (SPR) analysis for eCIRP and PS-OMe miR130 interaction

eCIRP was immobilized on the surface of sensor as a ligand and PS-OMe miR130 was injected as an analyte. Binding reactions were performed in PBS 0.05% P20, pH7.4. Carboxyl sensors were used for the experiments. The sensor was first cleaned by injection 10 mM HCl 150 µl, followed by injection of 150 µl of the mixture of 1 aliquot of *N*-ethyl-*N*′-[3-diethylaminopropyl]-carbodiimide (EDC) and 1 aliquot of *N*-hydroxysuccinimide (NHS) to activate the sensor surface. An aliquot of 200 µl of 50 µg/ml of the ligand diluted in 10 mM sodium acetate (pH 5) was injected into channel-2 of the sensor for immobilization. Next, 150 µl of 1 M ethanolamine (pH 8.5) was injected to deactivate the remaining active sites on channel 1&2. The channel-1 was used as a control to evaluate nonspecific binding. The binding analyses were performed at a flow rate of 40 µl per min at 20 °C. To evaluate the binding, the analyte ranging from 10 to 300 nM were injected into channel-1 & 2, and the real-time interaction data were analyzed by TraceDrawer (Nicoya). The signals from the control channel-1 were subtracted from the channel coated with the ligand-2 for all samples. Data were globally fitted for 1:1 binding (one-to-one model).

### Interaction between TLR4 and PS-OMe miR130 bound eCIRP

To determine the effect of PS-OMe miR130 on eCIRP binding to TLR4 or the TLR4/MD2 complex, NTA sensors were used. Human TLR4 & human TLR4/MD2 were purchased (R&D systems). The NTA sensor was first cleaned by injection 10mM HCL 150 µl and followed by injection of 150 µl of EDTA. Then the surface was activated by an injection of 40 mM NiCl_2_. Human TLR4 or the human TLR4/MD2 was immobilized in the running buffer at concentration 50 µg/ml to channel 2; eCIRP was injected as an analyte in concentrations of 125 nM to 1 µM. For effect of PS-OMe miR130, eCIRP was preincubated with PS-OMe miR130 with different concentration for 30 min at room temperature and then the complex was injected to channel 1 and 2. Binding reactions were carried out at 10 mM HEPES buffer, 150 mM NaCl, 3 mM EDTA, 0.05% P20, pH 7.4 at a flow rate of 40 µl/min at 20 °C. The channel-1 was used as a control to evaluate nonspecific binding, and the real-time interaction data were analyzed by TraceDrawer (Nicoya). The signals from the channel-1 were subtracted from the channel-2 coated with the ligand for all samples. Data were globally fitted for 1:1 binding (one-to-one model).

### Three-dimensional virtual modeling

#### Structure modeling

The nucleotide sequence of miRNA 130b-3p was derived from miRbase database (Kozomara et al. 2019). PS-OMe miR130 was modelled using PyMOL builder (PyMOL 2021). First, the three 5′ and 3′ terminal base in the phosphodiester linkage were replaced with sulfur giving rise to terminal phosphorothioate bonds. Second, 2′*O*-methyl ribose bases were incorporated throughout the miRNA.

#### Docking studies

The docking of CIRP and PS-OMe miR130 was performed using NPDock (Tuszynska et al. 2015), which combines GRAMM program to perform a rigid body global search, ranking, and scoring of best decoys using statistical potentials, clustering of best decoys. Finally, a Monte Carlo simulated annealing procedure (involving protein and nucleic acid molecules as rigid bodies) to optimize the protein-nucleic acid interactions in the representative clusters. The CIRP-PS-OMe miR130 and TLR4 receptor structure were docked using HDock (Yan et al. 2017), the FFT based translational search algorithm, which is optimized by iterative knowledge based scoring function, which is used both for protein-DNA and protein-RNA interactions and Patchdock (Schneidman-Duhovny et al. 2005), the algorithm that uses object recognition and image segmentation techniques. The interactions of CIRP and PS-OMe miR130 and the CIRP- PS-OMe miR130 and TLR4 structure were analyzed using PDBePISA tool (Krissinel et al. 2007). All the protein-microRNA structure complexes were visualized using PyMOL and Chimera tools (PyMOL 2021; Pettersen et al. 2004).

### Mouse model of cecal ligation and puncture (CLP)

CLP was performed as previously described (Denning et al. 2020; Gurien et al. 2020). Briefly, mice were anesthetized with 2–4% inhalation isoflurane and placed in the supine position. The ventral abdomen was shaved and then disinfected by swabbing with Betadine alternated two times with 70% alcohol. A 2 cm incision was made, and the cecum was exposed. The cecum was ligated with 4-0 silk suture 1 cm proximal to the distal end of the cecum. The cecum was then punctured twice with a 22-guage needle and a small amount of cecal contents was extruded from each puncture. The cecum was then placed back in the peritoneal cavity and the incision was closed in two layers; a subcutaneous bolus of 1 ml of normal saline, and a subcutaneous dose of buprenorphine (0.05 mg/kg) were given. Mice were allowed to recover from surgery and anesthesia and then returned to their home cages. After 20 h, mice were sacrificed, and blood and lung tissue were collected and stored at − 80 °C for quantitative analysis. A section of the right lower lobe of the lung was stored in 10% formalin for histologic analysis. Mice were randomly assigned to create a total of 3 group: sham, CLP + vehicle, and CLP + treatment.

### In vivo administration of PS-OMe miR130

After closure of the abdomen, vehicle (PBS) or PS-OMe miR130 at a dose of 12.5 nmol/mouse was injected intravenously via retroorbital injection using a 28G needle. The dose was determined from our previous study investigating the unmodified miRNA 130b-3p mimic in eCIRP induced inflammation and polymicrobial sepsis (Gurien et al. 2020).

### Measurement of organ injury markers

Whole blood samples were centrifuged at 3000×*g* for 10 min to collect serum, which was then stored at − 80 °C prior to use. Serum levels of LDH was determined using specific colorimetric enzymatic assays (Pointe Scientific, Canton, MI) according to manufacturer’s instructions.

### Cytokine measurements by enzyme-linked immunosorbent assay (ELISA)

Cell culture supernatant or mouse serum was analyzed by ELISA kits specific for IL-6, TNF-α (BD Biosciences, San Jose, CA), and IL-1β (Invitrogen, Thermofisher Scientific) according to manufacturer’s instructions.

### Histological evaluation of lung injury

Lung tissues were collected from the right lower lobe and were fixed in 10% formalin before being embedded in paraffin. Tissues were cut in 5 μm cuts and stained with hematoxylin-eosin. Slides were evaluated under light microscopy to evaluate the degree of lung injury. Scoring was performed using a system created by the American Thoracic Society (Matute-Bello et al. 2011). Scores ranged from 0 to 1 and were based on neutrophils in the alveolar space, neutrophils in the interstitial space, hyaline membranes, proteinaceous debris filling the airspaces, and alveolar septal thickening.

### Lung myeloperoxidase (MPO) assessment

Lung tissue was homogenized by sonication in 500 µl of potassium phosphate buffer containing 0.5% hexadecyltrimethylammonium bromide. Two freeze–thaw cycles were performed over dry ice. Samples were centrifuged to collect the supernatant. The protein concentration of the supernatant was determined. The reaction was then carried out in a 96-well plate by adding samples into phosphate buffer containing *O*-dianisidine hydrochloride and H_2_O_2_. Light absorbance was read at 460 nm over a period of 5 min. Results were normalized to the protein concentration of each sample. MPO activity (1 unit was equal to the change in absorbance per min) was expressed as units per gram of protein.

### Measurement of cytokines and chemokines by reverse transcription-quantitative (RT-qPCR) analysis

Total RNA was extracted from ischemic portions of the liver by TRIzol reagent (Invitrogen, Thermo Fisher Scientific Inc.) and was reverse transcribed into cDNA with reverse transcriptase (Applied Biosystems, Thermo Fisher Scientific Inc.). PCR reactions were carried out in 20 µl of a final volume of 0.08 µM of each forward and reverse primer, cDNA, water, and SYBR Green master mix (Applied Biosystems, Thermo Fisher Scientific Inc.). Amplification and analysis were conducted in a Step One Plus real-time PCR machine (Applied Biosystems, Thermo Fisher Scientific Inc.). Mouse β-actin mRNA was used as an internal control for amplification, and relative gene expression levels were calculated using 2^−ΔΔCt^ method. Relative expression of mRNA was expressed as a fold change in comparison with sham tissues. The sequence for the primers used are as follows:

- IL-6, 5′-CCGGAGAGGAGACTTCACAG-3′ (forward), 5′-CAGAATTGCCATTGCACAAC-3′ (reverse);
- TNF-α, 5′-AGACCCTCACACTCAGATCATCTTC-3′ (forward), and 5′-TTGCTACGACGTGGGCTACA-3′ (reverse);
- IL-1β, 5′-CAGGATGAGGACATGAGCACC-3′ (forward), and 5′-CTCGCAGACTCAAACTCCAC-3′ (reverse);
- KC, 5′-GCTGGGATTCACCTCAAGAA-3′ (forward), and 5′-ACAGGTGCCATCAGAGCAGT-3′ (reverse);
- MIP-2, 5′-CCCTGGTTCAGAAAATCATCCA-3′ (forward), and 5′-GCTCCTC-CTTTCCAGGTCAGT-3′ (reverse);
- β-Actin, 5′-CGTGAAAAGATGACCCAGATCA-3′ (forward), and 3′-TGGTACGACCAGAGGCATACAG-3′ (reverse).

### TUNEL assay

Apoptosis was assessed using terminal deoxynucleotidyl transferase dUTP nick end labeling (TUNEL) assay. For TUNEL staining, fluorescence staining was performed using a commercially available In Situ Cell Death Detection Kit (Roche Diagnostics, Indianapolis, IN). The assay was conducted according to the manufacturer’s instructions. 4′,6-Diamidino-2 phenylindole (DAPI) was used as a nuclear counterstain. TUNEL positive cells were counted using ImageJ software.

### Survival study

Animals also underwent a 10-day survival study as previously described (Denning et al. 2020). For the survival experiments, mice underwent CLP with a single puncture using 22G needle and were given 500 µl of the antibiotic imipenem (0.5 µg/kg, Merck) and 500 µl of normal saline subcutaneously at the time of laparotomy. The mice were then given a one-time dose of vehicle or PS-OMe miR130 (12.5 nmol/mouse) via retroorbital injection. Mice were then monitored twice daily for 10 days for their survival rates.

### Statistical analysis

Data represented in the figures are expressed as mean ± SEM, was checked for normality using Kolmogorov–Smirnov test. Normally distributed data was compared by using one-way analysis of variance (ANOVA) using Student–Newman–Keuls (SNK) post hoc analysis for multiple groups. When appropriate, non-normally distributed data was compared using nonparametric 1-way comparison among multiple groups using Kruskal–Wallis test with Dunn’s multiple-comparisons test. The specific tests used for each graph are identified in the figure legends. Survival rates were analyzed by the Kaplan–Meier estimator and compared using a log-rank test. Differences in values were considered significant if p ≤ 0.05. Data analysis was carried out using GraphPad Prism graphing and statistical software (GraphPad Software).

## Results

### PS-OMe miR130 reduces eCIRP mediated inflammation in vitro

**Fig. 1:**
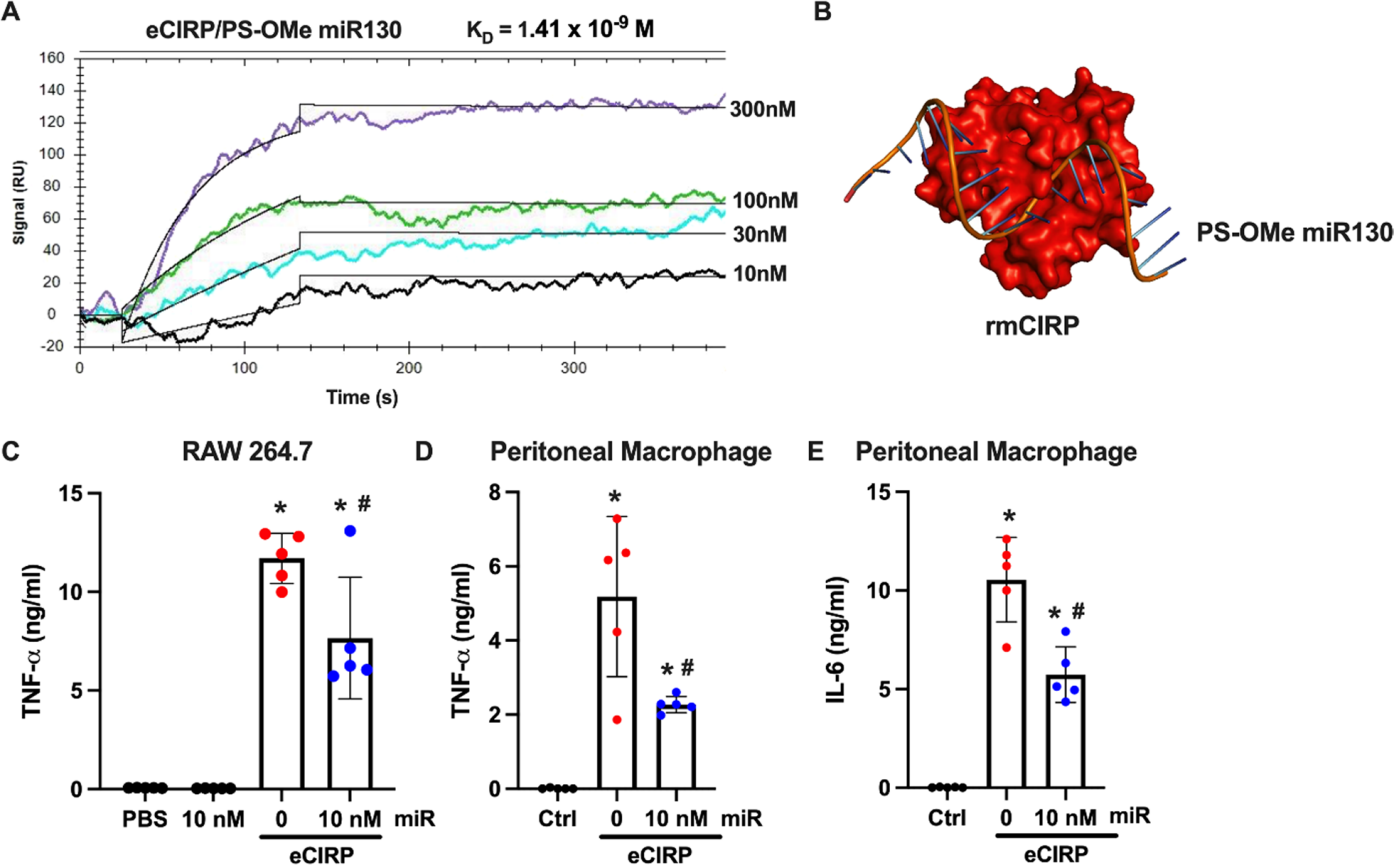
PS-OMe miR130 decreases eCIRP mediated inflammation in vitro.** A** Biotinylated PS-OMe miR130 was individually immobilized on a sensor chip SA, and eCIRP of varying concentrations was injected as the analyte (n = 3 independent SPR experiments). **B** Computational 3D-modeling illustrating docking of PS-OMe miR130 and eCIRP. RAW264.7 cells (1 × 10^6^ cells/ml) were treated with PBS (Ctrl), eCIRP (1 µg/ml) with 0 or 10 nM of PS-OMe mIR130 (miR). After 24 h, supernatant was collected and **C** TNF-α levels were measured. Mouse peritoneal macrophages (1 × 10^6^ cells/ml) were treated with PBS, eCIRP (1 µg/ml), or eCIRP (1 µg/ml) with 10 nM of PS-OMe miR130 (miR). After 24 h, supernatant was collected and **D** TNF-α and **E** IL-6 were measured (n = 5 replicates/group). Data expressed as means ± SD and compared by one-way ANOVA and SNK method (*P ≤ 0.05 versus Ctrl; ^#^P ≤ 0.05 vs. eCIRP)

First, the interaction between eCIRP and PS-OMe miR130 was evaluated by SPR analysis and the result showed a K_D_ of 1.41 × 10^−9^ M, indicating a very strong interaction (Fig. 1A). Further, the binding energy between eCIRP and PS-OMe miR130 using computational modeling was analyzed. PS-OMe miR130 binds strongly to eCIRP (binding energy = − 23.0 kcal/mol) (Fig. 1B), confirming the SPR data. The binding energy is a measure of the stability of the complex; if the value is more negative, this means that energy is released as the complex is formed and is more stable. If the value is more positive, it indicates that the complex requires energy to form and is less stable. Additionally, the effect of PS-OMe miR130 on eCIRP mediated inflammation was analyzed in vitro. eCIRP treatment in RAW 264.7 cells dramatically increased TNF-α levels whereas PS-OMe miR130 combined with eCIRP decreased TNF-α levels by 34.5% (Fig. 1C). Treatment with PS-OMe miR130 alone demonstrated no increase relative to PBS (Fig. 1C). In mouse peritoneal macrophages, both TNF-α and IL-6 showed significant increases after eCIRP treatment. These levels were reduced by 56.2% and 45.6% respectively after treatment (Fig. 1D, E). These data indicate that PS-OMe miR130 strongly binds to eCIRP and significantly decreases eCIRP mediated inflammation in vitro.

### PS-OMe miR130 is highly stable in vitro and in vivo

**Fig. 2:**
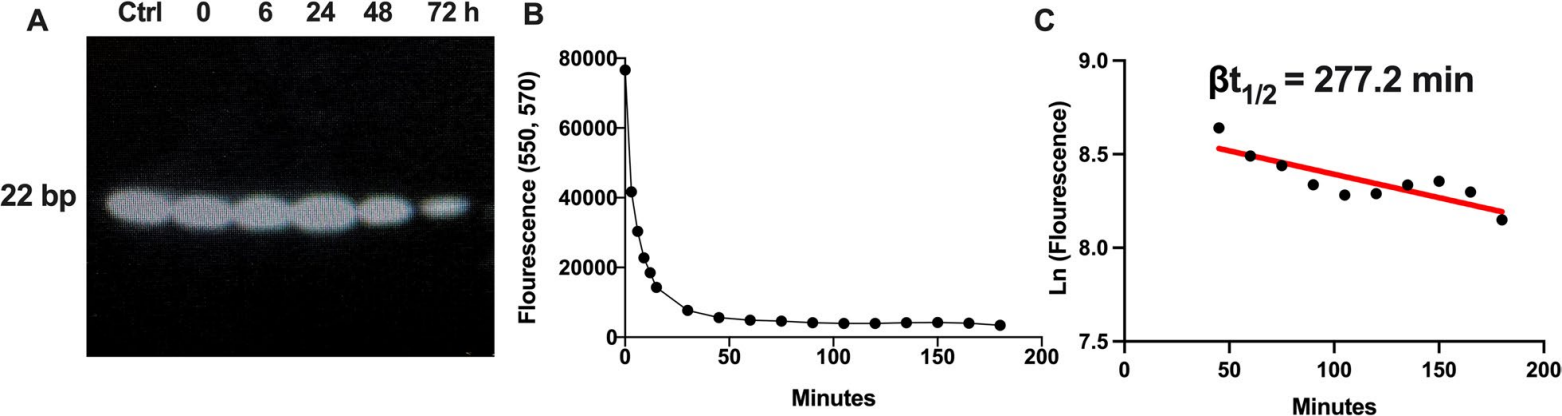
Stability of PS-OMe miR130 in vitro and in vivo. **A** 8 µM of PS-OMe miR130 was incubated in DMEM containing 10% non-heat-inactivated FBS for different time points. Samples were run on a denaturing gel and stained with ethidium bromide and viewed via UV light (n = 3 independent stability experiments). **B** In vivo half-life was determined by measuring the fluorescence in serum at different timepoints after injecting PS-OMe miR130 labeled with 3′ cy-3. Fluorescence was measured at each time point. **C** The natural logarithm (Ln) of the fluorescence was graphed over the elimination phase time-period. The slope was determined and using the slope, the elimination half-life was calculated (n = 4)

As oligonucleotides are known to be unstable, we first characterized the stability of PS-OMe miR130 in vitro. PS-OMe miR130 demonstrated stability in vitro as there was still a significant amount of the miRNA remaining after 72 h of incubation in 10% non-heat inactivated FBS (Fig. 2A). Further, we performed an in vivo half-life pharmacokinetic study that demonstrated the β half-life (elimination half-life) of PS-OMe miR130 to be 277.2 min, which shows significant stability after in vivo administration (Fig. 2B, C).

### PS-OMe miR130 blocks the interaction of eCIRP to TLR4

**Fig. 3:**
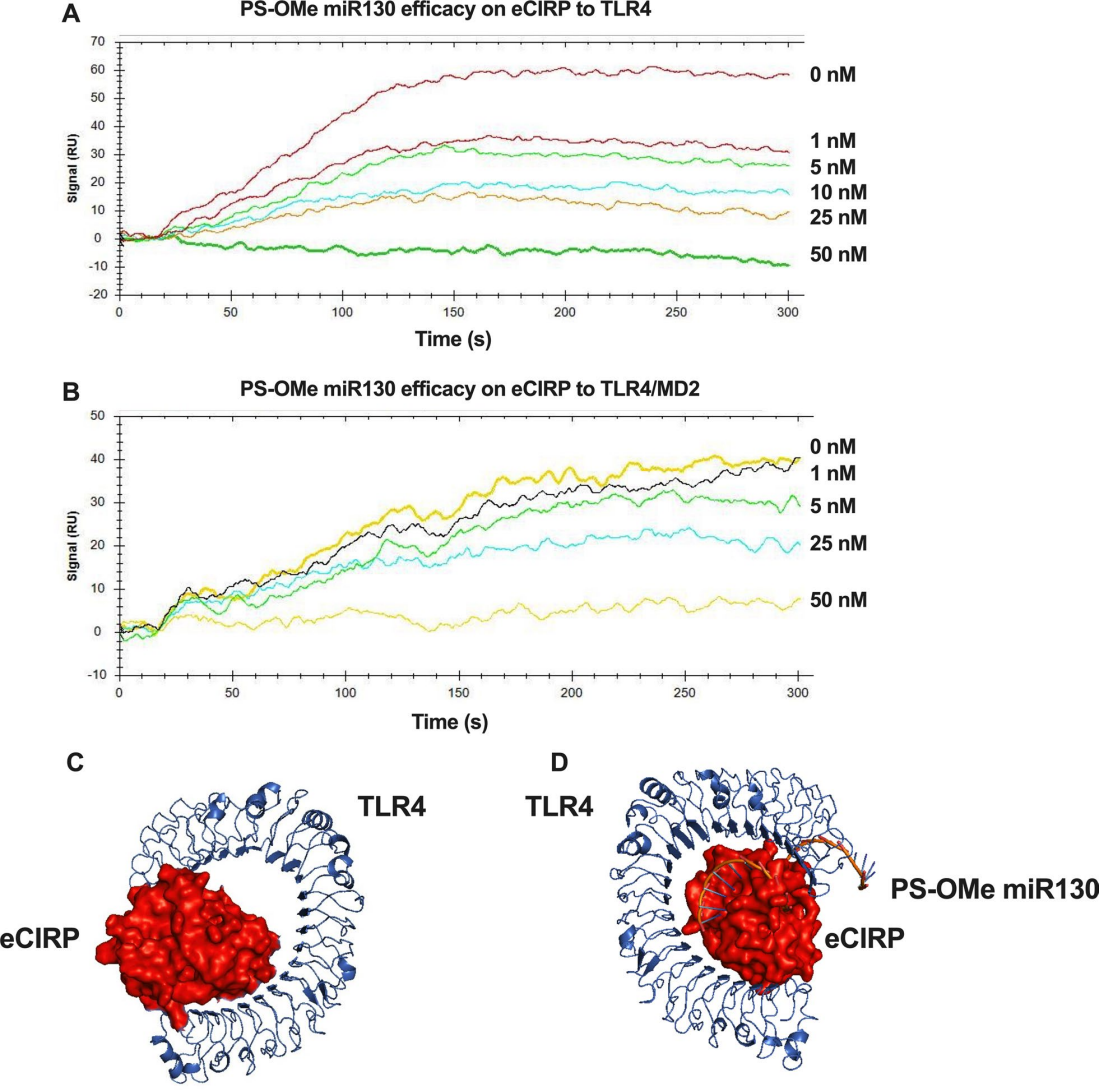
PS-OMe miR130 acts to decrease binding of eCIRP to TLR4. **A** Biotinylated TLR4 was individually immobilized on a sensor chip SA, and eCIRP (500 nM) incubated with various doses of PS-OMe miR130 (1–50 nM) in PBS was injected as the analyte. SPR analysis demonstrates a dose related decrease in binding affinity of eCIRP for TLR4 when combined with increasing doses of the engineered miRNA. The K_D_ decreased from 6.97 × 10^−8^ M at 0 nM of the miRNA to 1.39 × 10^−7^ M at 5 nM (n = 3 independent SPR experiments). **B** Biotinylated TLR4/MD2 was individually immobilized on a sensor chip SA, and eCIRP (1000 nM) incubated with various doses of the PS-OMe miR130 (1–50 nM) in PBS was injected as the analyte. SPR analysis demonstrates decreased binding affinity of eCIRP for the TLR4/MD2 complex when combined with increasing doses of PS-OMe miR130. The K_D_ decreased from 2.0 × 10^−8^ M with 0 nM of the miRNA to 1.13 × 10^−7^ with 5 nM of PS-OMe miR130 (n = 3 independent SPR experiments). **C** Computational modeling was performed between eCIRP and TLR4 and demonstrated that a strong binding complex is created. **D** Modeling was performed between a combined eCIRP/PS-OMe miR130 and TLR4 and demonstrates a less stable complex

We then wanted to determine the mechanism of action for PS-OMe miR130. TLR4 serves as the putative receptor for eCIRP (Qiang et al. 2013). As such, we determined the K_D_ value of eCIRP-TLR4 to be 6.97 × 10^−8^ M, indicating a strong interaction (Fig. 3A). SPR demonstrated that when incubating eCIRP with different doses of PS-OMe miR130, there was inhibition of eCIRP’s binding to TLR4, as noted by the decreasing K_D_ at 0 nM of PS-OMe miR130 (6.97 × 10^−8^ M) and at 5 nM of PS-OMe miR130 (6.12 × 10^−7^ M) (Fig. 3A). In fact, at 50 nM of PS-OMe miR130, there was complete inhibition of eCIRP to TLR4. As TLR4 is known to dimerize for downstream signaling and that MD-2 is required for dimerization (Park et al. 2009), SPR was also performed with the TLR4/MD2 complex and a similar result was observed. The K_D_ decreases from 2.00 × 10^−8^ M to 1.13 × 10^−7^ M at doses of 0 and 5 nM of PS-OMe miR130, respectively (Fig. 3B). There was no binding between PS-OMe miR130 and TLR4 or the TLR4/MD2 complex. Additionally, 3D computational modeling was performed to understand eCIRP’s relationship with TLR4. As expected eCIRP creates a stable binding complex with TLR4 (binding energy = − 8.1 kcal/mol) (Fig. 3C). Finally, computational modeling was performed with PS-OMe miR130 bound to eCIRP in relation to TLR4. Modeling demonstrates that the miRNA bound eCIRP creates a less stable complex with TLR4 (binding energy = 5.4 kcal/mol) (Fig. 3D). This in conjunction with the SPR data suggests that PS-OMe miR130 acts to decrease the binding of eCIRP and TLR4.

### PS-OMe miR130 decreases systemic injury and inflammation

**Fig. 4:**
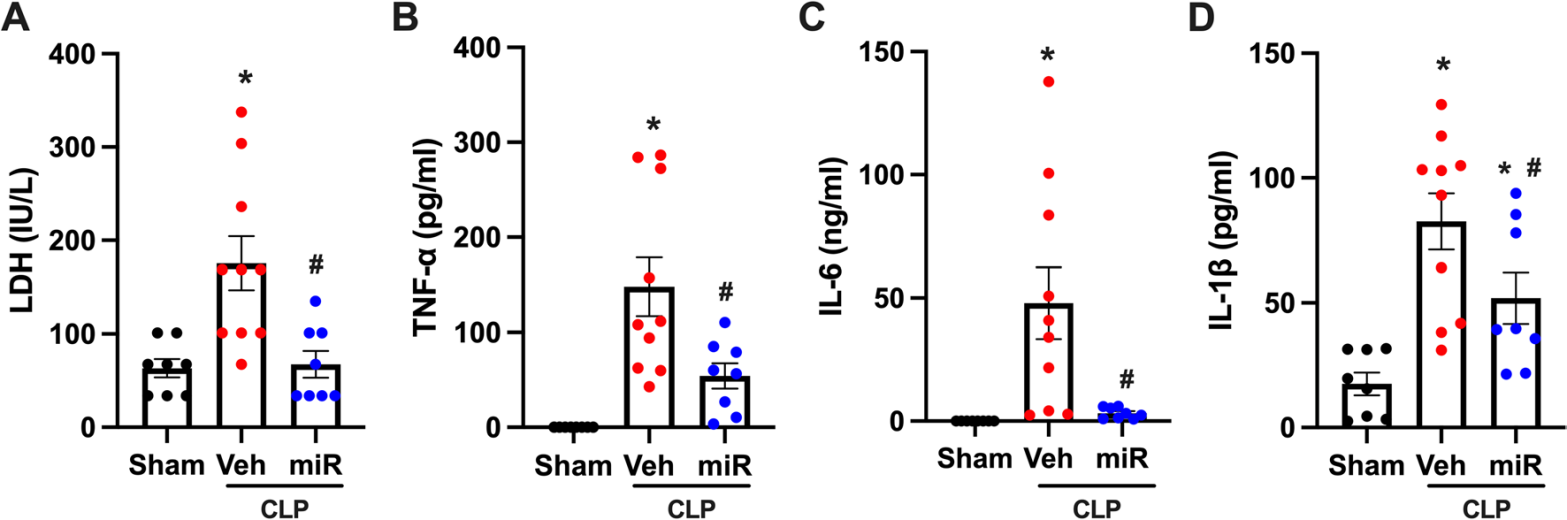
PS-OMe miR130 decreases systemic injury and inflammation. Blood was collected from sham, vehicle (veh), and PS-OMe miR130 (miR) treated mice at 20 h after CLP. **A** Serum LDH was determined using specific colorimetric enzymatic assays. Serum **B** TNF-α, **C** IL-6, and **D**  IL-1β were measured by ELISA (n = 8–10/group). For **A** data expressed as means ± SEM and compared by Kruskal–Wallis test with Dunn’s method (*P ≤ 0.05 versus sham; ^#^P ≤ 0.05 vs. veh). All other data expressed as means ± SEM and compared by one-way ANOVA and SNK method (*P ≤ 0.05 versus sham; ^#^P ≤ 0.05 vs. veh)

After, PS-OMe miR130 was tested in vivo in a murine model of polymicrobial sepsis as we confirm that PS-OMe miR130 does inhibit eCIRP mediated inflammation in vitro. First, systemic injury and inflammation was measured after CLP. LDH increased significantly in the serum in vehicle treated mice (Fig. 4A). A decrease of 62% was observed in PS-OMe miR130 treated mice demonstrating significantly less cellular injury after CLP. Next, markers of systemic inflammation including TNF-α, IL-6, and IL-1β were measured (Fig. 4B–D). In all three groups, a significant increase was demonstrated in vehicle treated mice. A reduction of 63%, 86%, and 37%, respectively, was observed after treatment showing a significant decrease in the degree of systemic inflammation.

### PS-OMe miR130 decreases lung inflammation and neutrophil infiltration

**Fig. 5:**
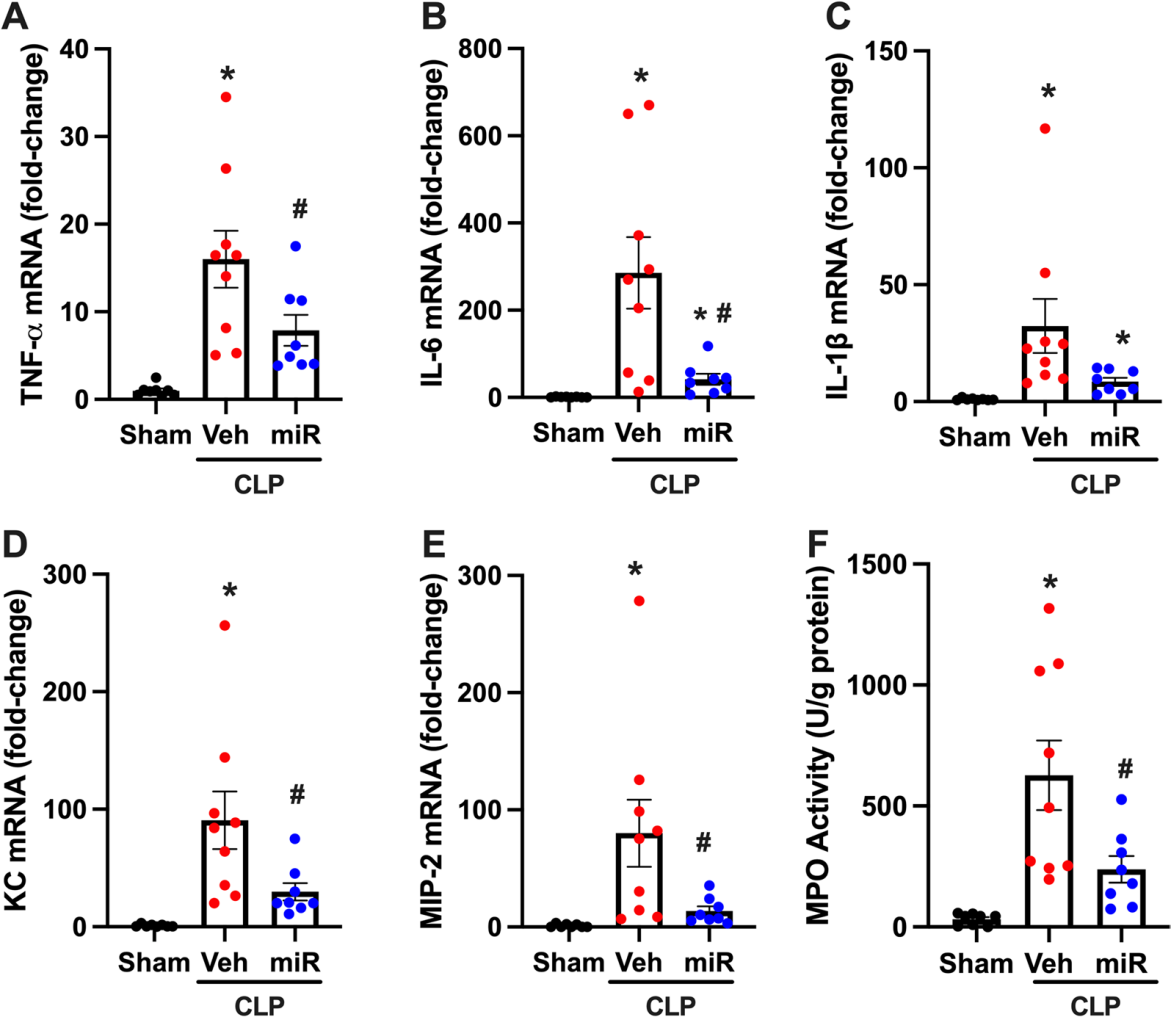
PS-OMe miR130 decreases lung inflammation and neutrophil infiltration. Lung tissue was collected for sham, vehicle (veh), and PS-OMe miR130 (miR) treated mice at 20 h after CLP. mRNA levels of **A** TNF-α, **B** IL-6, and **C** IL-1β, were measured by RT-qPCR. mRNA levels for **D** KC and **E** MIP-2 were assessed to determine chemokine expression in lung tissue. **F** Myeloperoxidase (MPO) activity was assessed spectrophotometrically. (n = 8–9/group). For **C** data expressed as means ± SEM and compared by Kruskal–Wallis test with Dunn’s method (*P ≤ 0.05 versus sham; ^#^P ≤ 0.05 vs. veh). All other data expressed as means ± SEM and compared by one-way ANOVA and SNK method (*P ≤ 0.05 versus sham; ^#^P ≤ 0.05 vs. veh)

After, inflammatory markers in the lung tissue were measured to determine the degree of acute lung injury and inflammation. First, TNF-α mRNA in lung tissue was measured and was found to be 16-fold higher in vehicle treated mice (Fig. 5A). PS-OMe miR130 treatment attenuated TNF-α levels by 51%. Further, IL-6 and IL-1β mRNA were measured in lung tissue (Fig. 5B, C). Increases of 286- and 32-fold were observed in vehicle treated mice. After treatment, these values decreased by 85% and 74% (p = 0.086) respectively. Next chemokine gene expression and neutrophil infiltration were assessed in lung tissue to further characterize inflammation in the lung. KC and MIP-2 mRNA levels increased by 91- and 132-fold respectively in vehicle treated mice (Fig. 5D, E) These levels reduced by 85% and 67% respectively in PS-OMe miR130 treated mice. After, MPO activity was determined to measure the degree of neutrophil infiltration (Fig. 5F). There was a significant increase in MPO activity in vehicle treated mice. PS-OMe miR130 significantly attenuated MPO activity levels, and a decrease of 62% was observed.

### PS-OMe miR130 attenuates lung injury and apoptosis

**Fig. 6:**
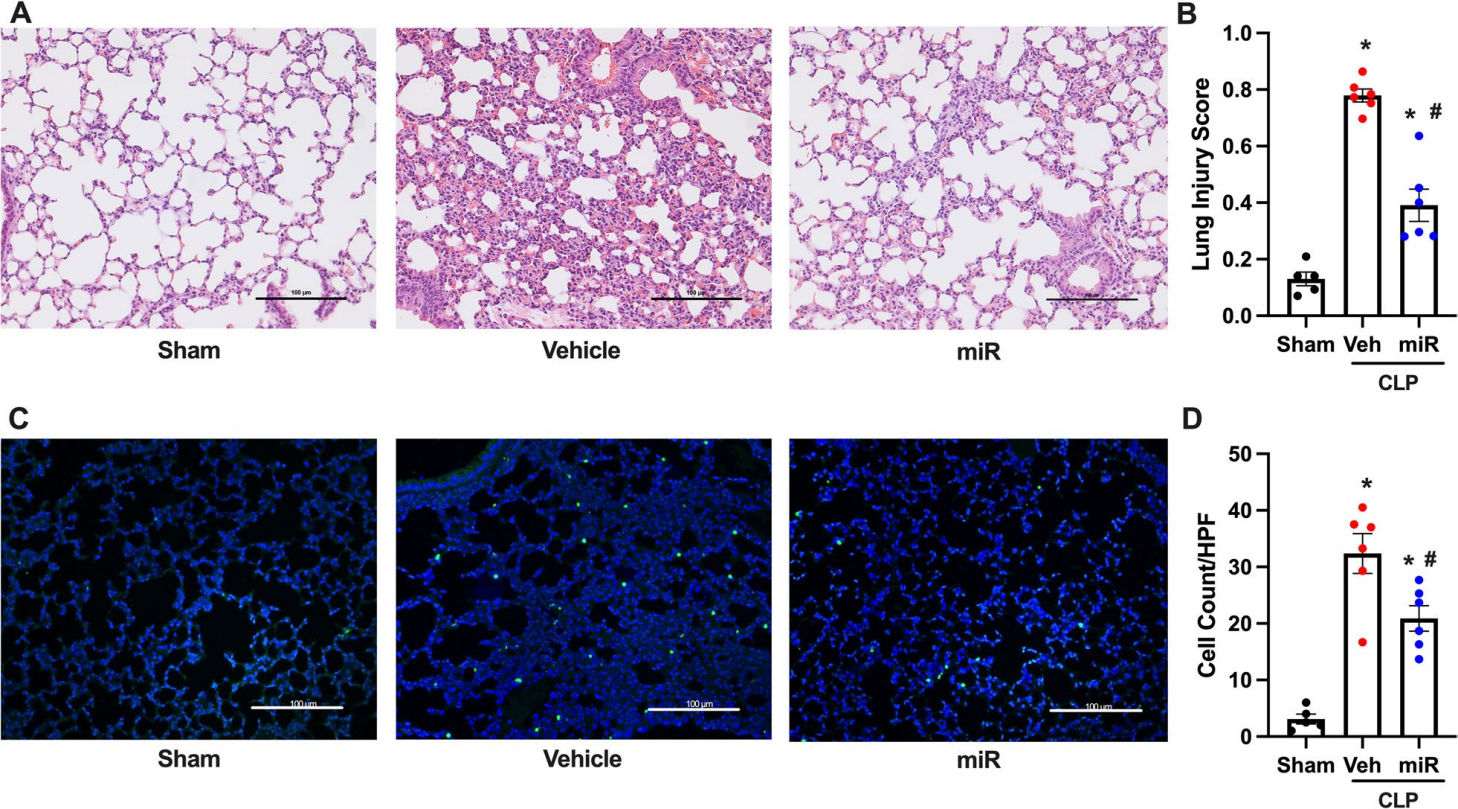
PS-OMe miR130 ameliorates lung injury. Lung tissue was collected for sham, vehicle (veh), and PS-OMe miR130 (miR) treated mice at 20 h after CLP. The right lower lobe was stored in 10% formalin. **A** Representative images of hematoxylin and eosin sections are shown at 200x. Scale bar 100 μm. **B** Lung injury was graded using a system created by the American Thoracic Society (n = 5–6/group). Sections were subjected to TUNEL assay to detect DNA fragmentation. TUNEL staining (green fluorescent) and nuclear counterstaining (blue fluorescent) of lung sections are shown. **C** Representative images of TUNEL staining from sham, vehicle (veh), and PS-OMe miR130 (miR) shown at ×200 magnification. **D** TUNEL positive cells were counted using ImageJ software (n = 5–6/group). Data expressed as means ± SEM and compared by one-way ANOVA and SNK method (*P ≤ 0.05 versus sham; ^#^P ≤ 0.05 vs. veh)

Lung injury was then assessed by histological scoring of H&E sections. Scoring was performed using criteria from the American Thoracic Society and ranged from 0 to 1 (Matute-Bello et al. 2011). The score is based on neutrophils in the alveolar space, neutrophils in the interstitial space, hyaline membranes, proteinaceous debris filling the airspaces, and alveolar septal thickening. Sham mice histology demonstrated normal lung tissue with open airways (Fig.6A). There was significant disruption of the normal lung architecture, neutrophil and cellular infiltration, and proteinaceous debris in the vehicle group. After PS-OMe miR130 treatment, some lung injury is still observed, however, to a much lesser degree. In the histological scoring, we observed a significant increase in the vehicle treated mice (Fig. 6B). Histology scoring was decreased by 50% after treatment. This indicated that PS-OMe miR130 protected mice from lung injury in CLP model of sepsis. Additionally, apoptosis was assessed in lung tissue after CLP via TUNEL staining. A significant increase in the amount of apoptosis is observed in the vehicle treated mice as there are visually more TUNEL positive cells in the vehicle sections (Fig. 6C). There is a clear decrease in the amount of TUNEL positive cells in the PS-OMe miR130 treated sections. TUNEL positive cells were counted and a reduction of 36% was observed in PS-OMe miR130 treated mice (Fig. 6D).

### PS-OMe miR130 improves survival in CLP-induced sepsis

**Fig. 7:**
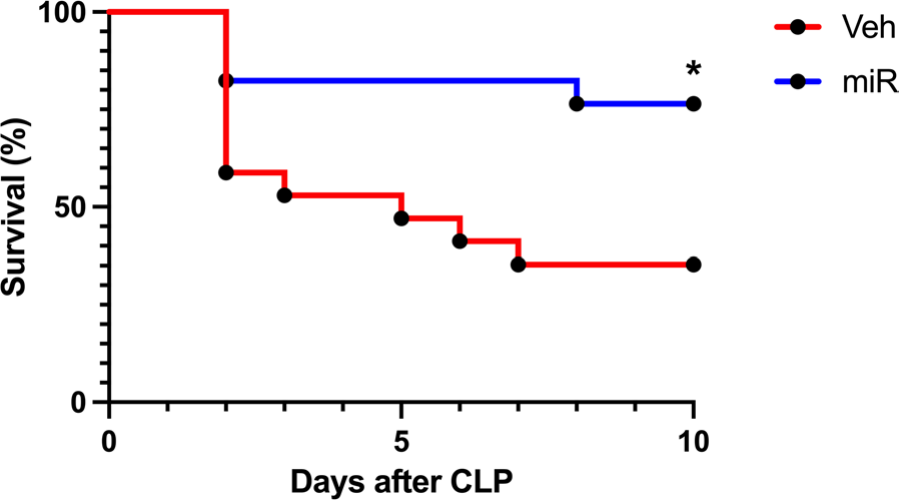
PS-OMe miR130 improves survival in sepsis. Mice were subjected to a survival CLP model of polymicrobial sepsis and were treated with either vehicle (veh) or PS-OMe miR130 (miR) (n = 17/group). Survival rates were measured over a 10-day period and were analyzed by the Kaplan–Meier estimator using a log rank test (*P < 0.05 versus veh)

Finally, survival was assessed after PS-OMe miR130 treatment to determine whether the treatment could improve the survival rate of septic mice (Fig. 7). In the 10-day survival study, 36% of the mice survived in the vehicle group while 76% survived in the treatment group indicating a significant net increase of 40% survival with PS-OMe miR130 treatment. Therefore, PS-OMe miR130 significantly improved survival after CLP induced polymicrobial sepsis.

## Discussion

Sepsis is an important clinical syndrome that is characterized by dysregulation of the immune system in response to an infection resulting in severe systemic inflammation, multisystem organ failure, and ultimately can result in death (Fleischmann et al. 2016; Seymour et al. 2016). In this study, we focus on a novel engineered miRNA based on miRNA 130b-3p as a potential therapeutic against sepsis. miRNA 130b-3p is a 22 base-pair miRNA that has been described in different disease states, which include different inflammatory conditions, hepatocellular carcinoma, bladder cancer, and lupus (Cui et al. 2016; Wang et al. 2015; Wang et al. 2014; Zheng et al. 2016). Our lab previously determined that miRNA 130b-3p is upregulated in sepsis conditions in both in humans and mice, and it can directly bind to eCIRP and inhibits eCIRP mediated inflammation in vitro and in vivo (Gurien et al. 2020). This study demonstrated the novel concept of how extracellular miRNAs can have impactful regulatory effects on inflammation. In the current study, we aimed to improve upon the original miRNA 130b-3p mimic as there are significant translational limitations to using naked RNA mimics as therapeutics, including poor oligonucleotide stability due to nuclease activity and renal elimination (Mitchell et al. 2008; Segal et al. 2020). To address these problems, chemical modifications were added to miRNA 130b-3p, which we called PS-OMe miR130, including 3 PS bonds to the 5′ and 3′ ends, and 2′OMe modifications throughout the mimic, to attempt to extend the therapeutics time in circulation. First, our goal was to prove that PS-OMe miR130 still retained the function of decreasing eCIRP mediated inflammation while improving stability. Then, PS-OMe miR130 was used in a murine model of polymicrobial sepsis to determine its role as a novel therapeutic. Our studies do in fact show that PS-OMe miR130 is highly stable, and functions by blocking the interaction between eCIRP and TLR4, which in turn reduced injury and inflammation, and improved survival in sepsis.

In the current study, we first wanted to determine whether the PS and 2′OMe chemical modifications added to miRNA 130b-3p would change its binding affinity to eCIRP. We show that PS-OMe miR130 has a very strong interaction with eCIRP via SPR and 3D computational modeling, which was similar to what was observed previously with miRNA 130b-3p (Gurien et al. 2020). Chemical modifications and delivery systems available for oligonucleotides to improve stability and attempt to enhance their effectiveness as therapeutics has been extensively studied (Mitchell et al. 2008; Segal et al. 2020; Behlke and Behlke 2008; Dasgupta et al. 2021; Dias and Stein 2002; Fu et al. 2019). A PS bond is the replacement of the bridging oxygen atom with a sulfur atom. They are used to protect the oligonucleotide from exonuclease activity and are generally used at the 5′ and 3′ ends of the oligonucleotide to minimize the amount of added bonds (Behlke and Behlke 2008; Lima et al. 2018). By minimizing the number of PS bonds, the unwanted effects of PS bonds are minimized, which includes binding to off target proteins, increased tissue uptake, and decreased binding to RNA targets (Behlke and Behlke 2008; Lennox et al. 2011; Lima et al. 2018; Crooke et al. 2020). 2′OMe bases are also used throughout the PS-OMe miR130. They are actually found on endogenous oligonucleotides, including mammalian ribosomal and transfer RNAs, and therefore does not present toxicity and are not immune stimulating (Behlke and Behlke 2008; Lennox et al. 2010; Lima et al. 2018). Importantly, both modifications have been used in combination by different groups in distinct animal models without observed toxicity (He et al. 2017; Krutzfeldt et al. 2005; Li et al. 2018).

Additionally, macrophages treated with eCIRP demonstrate a significant decrease in cytokine production after treatment with the PS-OMe miR130. We have previously shown that TLR4 is the first receptor identified for eCIRP on not only macrophages but also neutrophils (Qiang et al. 2013; Ode et al. 2018). In addition to macrophages, immune cells such as neutrophils, NK cells or T cells are certainly capable of responding to eCIRP and PS-OMe miR130 could decrease eCIRP-mediated inflammation in these cells. Additional studies with other immune cells can be addressed in the future. Importantly, this data demonstrates that PS-OMe miR130 can neutralize eCIRP’s inflammatory function in the extracellular environment, as eCIRP and PS-OMe miR130 were preincubated prior to cell treatment. This is an atypical use for miRNA-based therapeutics as many groups take advantage of the gene silencing properties of the miRNA and design antisense therapeutics with the goal of intracellular gene silencing (Dias and Stein 2002; Lima et al. 2018). Complex delivery systems have been designed with this goal mind (Dasgupta et al. 2021; Fu et al. 2019). Here we take advantage of how this specific miRNA has regulatory properties on an inflammatory DAMP, i.e., eCIRP to decrease inflammation. Further, there was no increase in cytokine levels with just the PS-OMe miR130 administration indicating that the miRNA is not pro-inflammatory. To summarize, these data suggest that the PS-OMe miR130 strongly binds to eCIRP and decreases eCIRP mediated inflammation in an in vitro system.

The in vitro and in vivo stability of PS-OMe miR130 were then tested as the goal of adding chemical modifications to the miRNA 130b-3p mimic was to improve its stability and half-life. In vitro, PS-OMe miR130 demonstrated significant stability after 72 h of incubation, which is comparable to what other groups have shown, as these chemical modifications are well known to increase the stability of oligonucleotide mimics (Barragan-Iglesias et al. 2018; Lennox et al. 2010). Although we haven’t measured the in vitro stability using immune cells, the in vivo half-life study is consisted of all cells in the body including the immune cells. In vivo, the elimination half-life or the slope of curve of the beta phase in circulation of PS-OMe miR130 was calculated to be 277.2 min (Fig. 2C) indicating the miRNA was highly stable in vivo. This value demonstrates the elimination half-life of PS-OMe miR130, which is a representation of the decrease in plasma concentration in the elimination phase. This does not acknowledge the distribution or the alpha phase of the therapeutic, which is calculated over the first 30 min of the study and is a measure of the rate of plasma distribution to tissues. Of note, the circulation half-life of a compound is usually determined by analyzing the beta phase or the clearance phase. Although we haven’t measured the levels of PS-Ome miR130 at 277.2 min or 4.6 h in the in vitro study, the levels obtained at 6 h indicate minimal degradation based on the visual image in gel electrophoresis (Fig. 2A, Lane 3). However, it is difficult to compare the levels between in vitro and in vivo because in in vivo, the injected miRNA could be trapped in the tissues such as the lungs, liver and/or the kidneys. Therefore, the miRNA present in circulation could be significantly lower than the actual amount in the body. Thus, the in vivo half-life appears to be a lot shorter than the in vitro assessment of the stability of the miRNA which indicates up to 72 h. Of note, this half-life study is limited and does not provide information regarding the amount of the PS-OMe miR130 present in tissue, and where it preferentially goes—for this, more rigorous half-life investigation which is ADME (absorption, distribution, metabolism, and excretion) studies will be needed to determine the biodistribution of PS-OMe miR130. This would be an interesting addition to our current work in the future as there are currently no studies to our knowledge that do this investigation with these specific oligonucleotide chemical modifications.

Additionally, we expand on the mechanism of action of PS-OMe miR130. In the past, our lab has extensively studied the role and relationship of eCIRP and TLR4 in both acute sterile and bacterial inflammation. eCIRP is well known be increased in sepsis conditions and to promote inflammation via binding to the TLR4/MD2 complex and displays a strong interaction to this complex (Aziz et al. 2019; Qiang et al. 2013). Further, this interaction is known to promote inflammation in sepsis (Qiang et al. 2013; Zhang et al. 2018). Additional studies have further demonstrated the importance of TLR4 signaling in sepsis, and that TLR4 is a well-established target for therapeutics (Kuzmich et al. 2017; Roger et al. 2009). Thus, by weakening or inhibiting the binding of eCIRP to TLR4, eCIRP-mediated inflammation will be decreased. Here we demonstrate decreased binding to TLR4 using two different modalities: SPR analysis, and computational modeling. Both SPR analysis and computational modeling clearly demonstrate that eCIRP’s binding to the TLR4 receptor decreases in the presence of PS-OMe miR130. Thus, subsequent inflammatory signaling will be diminished, shedding light on the possible mechanism for the novel therapeutic. Importantly, it should be reiterated that PS-OMe miR130 did not bind to TLR4 or TLR4/MD2 complex. In this study, we only check binding to TLR4 as TLR4 is thought to be the primary receptor for eCIRP, but for future investigations, it would be interesting to study how PS-OMe miR130 affects eCIRP binding its other receptors, including TREM-1 (Denning et al. 2020).

Finally, we tested PS-OMe miR130 as a novel therapeutic in a murine model of CLP-induced polymicrobial sepsis. We first demonstrate that there was significant protection in terms of systemic injury and inflammation. Additionally, we demonstrate decreased acute lung injury (ALI) after treatment via decreased lung cytokine/chemokine gene expression, neutrophil infiltration, lung injury, and apoptosis. This is important in terms of the translational potential of this therapeutic as patients with acute respiratory distress syndrome (ARDS) have significant morbidity and mortality (Gotts and Matthay 2016). Further, the finding of decreased neutrophil infiltration and apoptosis after treatment are critical as these events are crucial components of lung inflammation after sepsis (Grommes and Soehnlein 2011). Finally, we performed a survival study and demonstrated that PS-OMe miR130 significantly improves the survival rate after sepsis. Although we did not measure eCIRP levels in our study, we have previously shown that eCIRP is released into circulation in rats at 20 h after CLP and in hemorrhagic shock patients admitted to surgical intensive care units (Qiang et al. 2013). As described above, eCIRP levels are well known to be increased in sepsis, and by blocking eCIRP we demonstrate significantly less injury and inflammation. eCIRP levels will likely be decreased after PS-OMe miR130 treatment due to an indirect effect of having less inflammation. miRNA’s have previously been studied in sepsis with the goal of altering gene function or in the context of being extracellular biomarkers (Lee et al. 2020; Meng et al. 2019; Xie et al. 2019). Interestingly, in sepsis, many circulating miRNAs are either up or downregulated and may have important regulatory functions for protection or exacerbation of sepsis related injury and inflammation (Lee et al. 2020). However, in this study, we attempt use a miRNA mimic as a therapeutic in a novel way: we created a translatable and stable therapeutic based on miRNA 130b-3p to dampen eCIRP mediated inflammation in the extracellular space in sepsis. Although sepsis is a very complex immune process involving many DAMPs and molecules, our data are strongly promising PS-OMe miR130 could be developed as a therapeutic for sepsis and therefore warrants further studies for our therapeutic and other miRNA’s as therapeutics for sepsis. It would be unrealistic to think that a therapy based on single miRNA focused on a single DAMP would be enough as a therapy for sepsis. Our findings further points to the complexity of eCIRP-mediated inflammation and warrants additional future investigation to the mechanism of eCIRP-induced inflammation which is beyond the scope of this study.

However, our study has some limitations that need to be addressed. One limitation is that it can be speculated that PS-OMe miR130 may actually enter the cell and have off target effects (Behlke and Behlke 2008; Crooke et al. 2020). Although this is possible for the PS-OMe miR130 to enter the cell, groups that have used similar modifications actually include a 3′-cholesterol group as this allows the mimic to get intracellular more easily (Lennox et al. 2011; Lima et al. 2018). Further, complex delivery systems have been designed with intracellular entry and gene silencing in mind (Dasgupta et al. 2021; Fu et al. 2019), therefore, we believe this is minimized in our model. Negative side effects are not observed neither in vitro nor in vivo, however, more rigorous toxicity studies will need to be performed. Another limitation includes only investigating one version of the engineered miRNA 130b-3p. Many different chemical modifications exist to different oligonucleotides (Behlke and Behlke 2008; Khvorova et al. 2017; Lennox et al. 2011). We modelled PS-OMe miR130 based on previous work done by other groups (He et al. 2017; Krutzfeldt et al. 2005; Li et al. 2018), however, further investigation of different combinations of chemical modifications may lead to a more improved engineered miRNA. Finally, in our model of sepsis we only explore one dose and timepoint for dosing of PS-OMe miR130. Different doses of the mimic would provide valuable therapeutic information. It is usually not possible to treat patients with sepsis therapies at the time of the initial insult. Therefore, post-treatment results are often necessary to evaluate the potential of a sepsis therapeutic. However, we did not perform a delayed experiment as we hypothesized that the chemical modifications on the PS-OMe miR130 would allow it to remain in circulation for longer periods of time. Our pharmacokinetic studies showed that the elimination half-life for PS-OMe miR130 is about 4.6 h suggesting that at least half of the injected amount of PS-OMe miR130 would still be present up to 4 h after CLP which would have been the time point chosen for post-treatment in a 20 h sepsis study. In fact, with one dose we were able to a show a reduction in injury and inflammation, and improvement in survival, and therefore we did not conduct a post-treatment study. Additional dose and time course studies can be done in the future to develop PS-OMe-miR130 as a therapy for sepsis. We also fully acknowledge that the animal models of sepsis have limitations and they may not represent the high complexity of the sepsis condition in humans.

## Conclusion

**Fig. 8:**
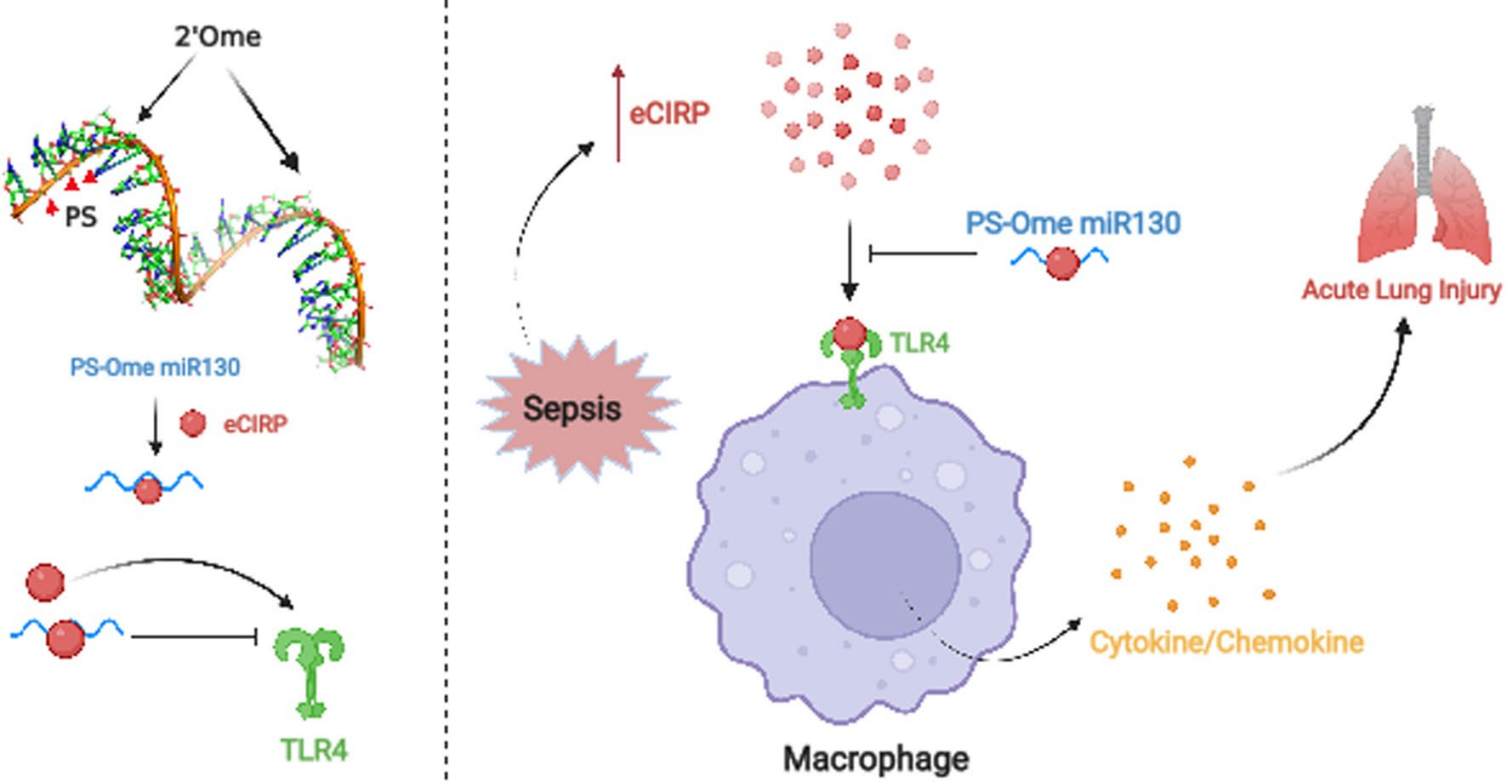
Schematic showing the engineered PS-OMe miR130 and its proposed mechanism for protection in sepsis. The modified miRNA 130b-3p mimic named PS-OMe miR130 binds to eCIRP and blocks eCIRP binding to TLR4. During sepsis, PS-OMe miR130 acts to block the interaction between eCIRP and its receptor TLR4, thereby attenuating inflammatory cytokine release and preventing acute lung injury

We synthesized PS-OMe miR130, an engineered miRNA 130b-3p, to improve upon the unmodified miRNA 130b-3p by enhancing the stability of the oligonucleotide. We first show that PS-OMe miR130 still binds to eCIRP and decreases eCIRP mediated inflammation in vitro. We then provide evidence demonstrating the stability of PS-OMe miR130 in vitro and in vivo. Further, we provide evidence that shows that PS-OMe miR130 acts by decreasing the binding of eCIRP to TLR4 receptor. Finally, we demonstrate the use of PS-OMe miR130 in a murine model of polymicrobial sepsis demonstrating lower levels of both systemic and local injury and inflammation, and improved survival (Fig. 8). Therefore, our study demonstrates the potential therapeutic uses of the engineered miRNA 130b-3p in sepsis and the novel use of chemically modified miRNA mimics as extracellular therapeutics in acute inflammation.

## Data Availability

All data generated or analyzed during this study are included in this article.

## Abbreviations

eCIRP: Extracellular cold inducible RNA binding protein
DAMP: Damage associated molecular pattern
TLR4: Toll-like receptor-4
TREM-1: Triggering receptor expressed on myeloid cells-1
PS: Phosphorothioate bonds
2′OMe: 2′ *O*-Methyl ribose modifications
SPR: Surface plasmon resonance
MD2: Myeloid differentiation factor-2
CLP: Cecal ligation and puncture
MPO: Myeloperoxidase
LDH: Lactate dehydrogenase
TUNEL: Terminal deoxynucleotidyl transferase dUTP nick end labeling

## References

[CR1] Aziz M, Brenner M, Wang P (2019). Extracellular CIRP (eCIRP) and inflammation. J Leukoc Biol.

[CR2] Barragan-Iglesias P, Lou TF, Bhat VD, Megat S, Burton MD, Price TJ (2018). Inhibition of poly(A)-binding protein with a synthetic RNA mimic reduces pain sensitization in mice. Nat Commun.

[CR3] Behlke MA (2008). Chemical modification of siRNAs for in vivo use. Oligonucleotides.

[CR4] Borjas T, Jacob A, Yen H, Patel V, Coppa GF, Aziz M (2022). Inhibition of the interaction of TREM-1 and eCIRP attenuates inflammation and improves survival in hepatic ischemia/reperfusion. Shock.

[CR5] Cen C, Yang WL, Yen HT, Nicastro JM, Coppa GF, Wang P (2016). Deficiency of cold-inducible ribonucleic acid-binding protein reduces renal injury after ischemia–reperfusion. Surgery.

[CR6] Crooke ST, Vickers TA, Liang XH (2020). Phosphorothioate modified oligonucleotide–protein interactions. Nucleic Acids Res.

[CR7] Cui X, Kong C, Zhu Y, Zeng Y, Zhang Z, Liu X (2016). miR-130b, an onco-miRNA in bladder cancer, is directly regulated by NF-kappaB and sustains NF-kappaB activation by decreasing cylindromatosis expression. Oncotarget.

[CR8] Dasgupta I, Chatterjee A (2021). Recent advances in miRNA delivery systems. Methods Protoc.

[CR9] Denning NL, Aziz M, Ochani M, Prince JM, Wang P (2020). Inhibition of a triggering receptor expressed on myeloid cells-1 (TREM-1) with an extracellular cold-inducible RNA-binding protein (eCIRP)-derived peptide protects mice from intestinal ischemia–reperfusion injury. Surgery.

[CR10] Denning NL, Aziz M, Murao A, Gurien SD, Ochani M, Prince JM (2020). Extracellular CIRP as an endogenous TREM-1 ligand to fuel inflammation in sepsis. JCI Insight.

[CR11] Dias N, Stein CA (2002). Antisense oligonucleotides: basic concepts and mechanisms. Mol Cancer Ther.

[CR12] Fleischmann C, Scherag A, Adhikari NK, Hartog CS, Tsaganos T, Schlattmann P (2016). Assessment of global incidence and mortality of hospital-treated sepsis. Current estimates and limitations. Am J Respir Crit Care Med.

[CR13] Fu Y, Chen J, Huang Z (2019). Recent progress in microRNA-based delivery systems for the treatment of human disease. ExRNA.

[CR14] Godwin A, Yang WL, Sharma A, Khader A, Wang Z, Zhang F (2015). Blocking cold-inducible RNA-binding protein protects liver from ischemia–reperfusion injury. Shock.

[CR15] Gotts JE, Matthay MA (2016). Sepsis: pathophysiology and clinical management. BMJ.

[CR16] Grommes J, Soehnlein O (2011). Contribution of neutrophils to acute lung injury. Mol Med.

[CR17] Gurien SD, Aziz M, Jin H, Wang H, He M, Al-Abed Y (2020). Extracellular microRNA 130b-3p inhibits eCIRP-induced inflammation. EMBO Rep.

[CR18] He Q, Wang F, Honda T, Lindquist DM, Dillman JR, Timchenko NA (2017). Intravenous miR-144 inhibits tumor growth in diethylnitrosamine-induced hepatocellular carcinoma in mice. Tumour Biol.

[CR19] Khvorova A, Watts JK (2017). The chemical evolution of oligonucleotide therapies of clinical utility. Nat Biotechnol.

[CR20] Kozomara A, Birgaoanu M, Griffiths-Jones S (2019). miRBase: from microRNA sequences to function. Nucleic Acids Res.

[CR21] Krissinel E, Henrick K (2007). Inference of macromolecular assemblies from crystalline state. J Mol Biol.

[CR22] Krutzfeldt J, Rajewsky N, Braich R, Rajeev KG, Tuschl T, Manoharan M (2005). Silencing of microRNAs in vivo with ‘antagomirs’. Nature.

[CR23] Kuzmich NN, Sivak KV, Chubarev VN, Porozov YB, Savateeva-Lyubimova TN, Peri F (2017). TLR4 signaling pathway modulators as potential therapeutics in inflammation and sepsis. Vaccines.

[CR24] Lagu T, Rothberg MB, Shieh MS, Pekow PS, Steingrub JS, Lindenauer PK (2012). Hospitalizations, costs, and outcomes of severe sepsis in the United States 2003 to 2007. Crit Care Med.

[CR25] Lee LK, Medzikovic L, Eghbali M, Eltzschig HK, Yuan X (2020). The role of microRNAs in acute respiratory distress syndrome and sepsis, from targets to therapies: a narrative review. Anesth Analg.

[CR26] Lennox KA, Behlke MA (2010). A direct comparison of anti-microRNA oligonucleotide potency. Pharm Res.

[CR27] Lennox KA, Behlke MA (2011). Chemical modification and design of anti-miRNA oligonucleotides. Gene Ther.

[CR28] Li J, Cai SX, He Q, Zhang H, Friedberg D, Wang F (2018). Intravenous miR-144 reduces left ventricular remodeling after myocardial infarction. Basic Res Cardiol.

[CR29] Lima JF, Cerqueira L, Figueiredo C, Oliveira C, Azevedo NF (2018). Anti-miRNA oligonucleotides: a comprehensive guide for design. RNA Biol.

[CR30] Matute-Bello G, Downey G, Moore BB, Groshong SD, Matthay MA, Slutsky AS (2011). An official American thoracic society workshop report: features and measurements of experimental acute lung injury in animals. Am J Respir Cell Mol Biol.

[CR31] Meng L, Cao H, Wan C, Jiang L (2019). MiR-539-5p alleviates sepsis-induced acute lung injury by targeting ROCK1. Folia Histochem Cytobiol.

[CR32] Mitchell PS, Parkin RK, Kroh EM, Fritz BR, Wyman SK, Pogosova-Agadjanyan EL (2008). Circulating microRNAs as stable blood-based markers for cancer detection. Proc Natl Acad Sci USA.

[CR33] Nishiyama H, Itoh K, Kaneko Y, Kishishita M, Yoshida O, Fujita J (1997). A glycine-rich RNA-binding protein mediating cold-inducible suppression of mammalian cell growth. J Cell Biol.

[CR34] Ode Y, Aziz M, Wang P (2018). CIRP increases ICAM-1(+) phenotype of neutrophils exhibiting elevated iNOS and NETs in sepsis. J Leukoc Biol.

[CR35] Park BS, Song DH, Kim HM, Choi BS, Lee H, Lee JO (2009). The structural basis of lipopolysaccharide recognition by the TLR4-MD-2 complex. Nature.

[CR36] Pettersen EF, Goddard TD, Huang CC, Couch GS, Greenblatt DM, Meng EC (2004). UCSF Chimera—a visualization system for exploratory research and analysis. J Comput Chem.

[CR44] PyMOL (version 2.5). 2021. https://pymol.org/.

[CR37] Qiang X, Yang WL, Wu R, Zhou M, Jacob A, Dong W (2013). Cold-inducible RNA-binding protein (CIRP) triggers inflammatory responses in hemorrhagic shock and sepsis. Nat Med.

[CR38] Rhee C, Jones TM, Hamad Y, Pande A, Varon J, O’Brien C (2019). Prevalence, underlying causes, and preventability of sepsis-associated mortality in US acute care hospitals. JAMA Netw Open.

[CR39] Roger T, Froidevaux C, Le Roy D, Reymond MK, Chanson AL, Mauri D (2009). Protection from lethal gram-negative bacterial sepsis by targeting toll-like receptor 4. Proc Natl Acad Sci USA.

[CR40] Schneidman-Duhovny D, Inbar Y, Nussinov R, Wolfson HJ (2005). PatchDock and SymmDock: servers for rigid and symmetric docking. Nucleic Acids Res.

[CR41] Segal M, Slack FJ (2020). Challenges identifying efficacious miRNA therapeutics for cancer. Expert Opin Drug Discov.

[CR42] Seymour CW, Liu VX, Iwashyna TJ, Brunkhorst FM, Rea TD, Scherag A (2016). Assessment of clinical criteria for sepsis: for the third international consensus definitions for sepsis and septic shock (sepsis-3). JAMA.

[CR43] Shankar-Hari M, Phillips GS, Levy ML, Seymour CW, Liu VX, Deutschman CS (2016). Developing a new definition and assessing new clinical criteria for septic shock: for the third international consensus definitions for sepsis and septic shock (sepsis-3). JAMA.

[CR45] Tuszynska I, Magnus M, Jonak K, Dawson W, Bujnicki JM (2015). NPDock: a web server for protein-nucleic acid docking. Nucleic Acids Res.

[CR46] Wang WY, Zhang HF, Wang L, Ma YP, Gao F, Zhang SJ (2014). High expression of microRNA-130b correlates with poor prognosis of patients with hepatocellular carcinoma. Diagn Pathol.

[CR47] Wang W, Mou S, Wang L, Zhang M, Shao X, Fang W (2015). Up-regulation of serum MiR-130b-3p level is associated with renal damage in early lupus nephritis. Sci Rep.

[CR48] Xie J, Zhang L, Fan X, Dong X, Zhang Z, Fan W (2019). MicroRNA-146a improves sepsis-induced cardiomyopathy by regulating the TLR-4/NF-kappaB signaling pathway. Exp Ther Med.

[CR49] Yan Y, Zhang D, Zhou P, Li B, Huang SY (2017). HDOCK: a web server for protein–protein and protein-DNA/RNA docking based on a hybrid strategy. Nucleic Acids Res.

[CR50] Zhang F, Brenner M, Yang WL, Wang P (2018). A cold-inducible RNA-binding protein (CIRP)-derived peptide attenuates inflammation and organ injury in septic mice. Sci Rep.

[CR51] Zheng H, Dong X, Liu N, Xia W, Zhou L, Chen X (2016). Regulation and mechanism of mouse miR-130a/b in metabolism-related inflammation. Int J Biochem Cell Biol.

